# Histories of violence among clients seeking substance use disorder treatment: a systematic mapping review

**DOI:** 10.3389/fpsyt.2024.1307641

**Published:** 2024-03-05

**Authors:** Sara Beeler, Olivia Gerrish, B. Grantham Aldred, Autumn Asher BlackDeer

**Affiliations:** ^1^ Jane Addams College of Social Work, University of Illinois Chicago, Chicago, IL, United States; ^2^ University Library, University of Illinois Chicago, Chicago, IL, United States; ^3^ Graduate School of Social Work, University of Denver, Denver, CO, United States

**Keywords:** substance misuse, trauma, systematic mapping review, treatment outcomes, integrated treatment, drug and alcohol, violence, substance use disorder

## Abstract

**Introduction:**

There is a limited literature base regarding the intersection of drug and alcohol treatment, violence, and trauma. While research substantiates that exposure to violence and trauma impacts the propensity to misuse substances, the conceptualization in clinical trials and practice has largely been narrow and gendered, referring only to intimate partner or domestic violence. Our systematic mapping review explored a more inclusive and expansive review of survivors of and perpetrators of violence and trauma (e.g., intimate partner violence, sexual assault, stalking, child abuse, political and community violence, criminal violence, micro violence, structural violence, and oppression) to establish: 1) the types of treatment settings included in intervention studies, 2) the common indicators of success or common outcomes recorded, and 3) understanding who is seeking treatment for drug and alcohol use with histories of violence.

**Methods:**

A systematic mapping review was conducted to identify any peer-reviewed articles published from 2011 to 2022. The Web of Science database was searched using a broad set of Boolean search terms related to violence, substance use disorders, and treatment. Over 8,800 records were identified from the systematic review with a total of 48 articles meeting inclusion criteria.

**Results:**

Most studies in this review included populations reporting perpetration of violence (n=23, 48%) versus participants reporting survival of trauma/violence (n=17, 35%). Results also indicated female identifying populations (n=19; 40%) were predominantly served, were treated in the US (n=33; 69%) and seen in an outpatient setting (n=24; 50%). Authors also were attentive to studies that included sexual and gender minorities and discovered only three studies (6%) explicitly acknowledging inclusion of transgender participants or participants in relationship with partners of the same sex; three more studies (6%) were focused on participants with histories of or engaging in sex work.

**Discussion:**

This review outlines treatment and research implications directly situated in the gap of service delivery found in this review. Specifically, the results elucidate the impact on minoritized and excluded identities based on gender, sexual preference, criminal legal status and directions for research and treatment to increase inclusion, representation, and equity across research and treatment settings.

## 1 Introduction

Substance use disorders (SUD) continue to be highly treated in the mental health field (1–4). Violence and substance use have been linked in many ways, especially concerning the use of substances has and perpetration of violence (5, 6). Emergent research has noted that the bidirectionality of substance use and violence also leads to increased violence victimization (5, 7–9). For example, Gilbert et al. (10) found that crack cocaine use leads to increased sexual and intimate partner violence. One study found that 40-60% of partnered individuals experienced a traumatic incident, in this case, intimate partner violence, within one year of entering substance use treatment (11).

Duke and colleagues (2018) found that alcohol use increased the victimization rate of violence in general. This finding is echoed in women as they are more likely to experience interpersonal violence (6). Cheng & Lo (12) identified that women who experience domestic violence seek treatment for substance use at higher rates than women that have not experienced domestic violence. It has been shown that exposure to violence and trauma directly impacts one’s likelihood to misuse substances (11, 13–16).

While the discussion around violence perpetration, victimization, and substance use treatment continues to be explored, less emphasis has been given to the intersection of violence and substance use treatment. Capezza et al. (5) described the lack of awareness surrounding the interconnected natures of substance use and intimate partner violence as the “largest obstacle” facing healthcare providers treating substance use. A continuous practice gap occurs as educational systems offer minimal resources to aid practitioners in addressing the complex interplay of substance use and histories of violence (17). Minimal measures have been made to address histories of violence in substance use treatment (5). Timko et al. (18) identified that a mere 16% of substance use treatment facilitated include intimate partner violence screenings. Capezza et al. (5) sampled 13,696 substance-use facilities and found that only 38.4% offered services related to intimate partner violence. Recent research suggests this number is relatively unchanged as they found general prevalence rates for violence screenings as low as 42% among a sample of the birthing population (19).

Facilities that provided substance use treatment in conjunction with violence resources were found to be inconsistent. Capezza et al. (5) assessed the prevalence of violence and substance use treatment by exploring intimate partner and sexual violence service provision in substance use treatment centers. Variability was found in agencies that provided co-occurring service options. In the above study, it was found that Indigenous-based treatment services were the most likely to provide violence intervention, while solo practices, religiously affiliated healthcare centers and hospitals generally provided violence treatment options at a much lower rate (5). A recent review from Romo-Avilés and colleagues (20) documented under 20% of articles addressed gender based violence in studies offering addiction treatment, even though almost 60% of articles document the effects of violence seen among the populations served in treatment for drugs and alcohol. Almost two decades of research continually call out limited research that shows integrated treatment despite the clear link between violence and substance use.

Furthermore, there is limited knowledge regarding where individuals with histories of violence and substance use are accessing treatment and its impact on outcomes. Recent research highlights that the COVID-19 pandemic decreased in person treatment access due to safety procedures, however virtual options, like telemedicine and other web-based appointments, have shown useful for initiation of medication assisted treatment for opioid use (21). National surveys continually show emergency departments are highly utilized among persons with SUD, particularly for opioid use, suggesting acute care is accessed (22), however little is known on where individuals are accessing chronic care, or treatment. Thus, researchers have repeatedly called for a review of integrated substance use and violence treatment (3, 4, 23–25).

Moreover, intergenerational trauma and substance use, influenced by systemic oppression, create a complicated landscape of treatment needs (26). Substance use and violence-integrated treatment are of critical importance to mental health professionals (18, 27) as we know that people are experiencing a variety of different forms and types of violence, from people we know, to those we do not, and from societal structures and formal systems from which we operate in daily (i.e., work, school, hospitals, community surveillance; 28). The lack of recognition of this broad experience and interaction of violence acts as an additional barrier to needed care and adds an additional complex layer to under-resourced, marginalized, and/or historically excluded populations (18). A growing awareness of systemic violence is experienced among Black, Indigenous, and communities of color (29). A disproportionate incarceration rate for violent crime and drug felonies calls for a closer look at how patriarchal, misogynistic, and racist systems impact the victimization of women, particularly Black women (30). Similarly, the systemic nature of racism and white supremacy has proven to have negative impacts on mental health and the misuse of substances in communities of color (28). Recent work has highlighted the need for the increased attention to white supremacy and racial discrimination in substance use treatment (26, 28).

Goldstein (31) described a conceptual framework that describes three ways in which violence and drugs (e.g. substances) interact, including psychopharmological violence, systemic violence, and economic compulsive violence. For example, the literature more largely explains the connection between drugs and alcohol and intimate partner violence (i.e. Goldstein’s “psychopharmacological violence”, p. 494), along with a smaller, yet growing body of knowledge highlighting the connection of substances and broader categories of violence. Goldstein’s (31) models capture this broader violence intersection as both “economic compulsive violence” (p. 496) and “systemic violence” (p. 497). Both parts of the model showcase how individuals can engage in violence that is driven to gain finances to obtain substances (e.g. armed/robbery or assault; economically motivation violence) and/or that violence is a naturally occurring phenomena that is intimately connected to substances (e.g. systemic violence; 31). The effects of mis/using drugs and alcohol use document harms across social (i.e. society and views of stigma), legal (i.e. arrest, incarceration, or community corrections), and economic (i.e. cost of use and potential loss of income via employment) structures (22, 32). Ultimately, Goldstein’s (31) framework highlights that the relationship between substances and violence is complex and can span all categories of violence, from micro (i.e. individual experiences of violence, rape or intimate partner violence) to macro (i.e. violence experienced at a population or neighborhood level, including gun violence, health disparities, or arrest). Relatedly, Lang and colleagues (33) scoping review found an opportunity to research the connection of sociopolitical factors and structural forces, like violence (i.e. oppression) across macro systems (e.g. healthcare, education, and housing), on interventions to address the consequences of substance use, including opioid overdose deaths (33). While dated, Goldstein’s (31) tripartite framework is the seminal research connecting violence and substance mis/use, differences in how violence is experienced among males and females, and is still applied to relevant research today (34).

Pertaining to terminology, this paper acknowledges ‘SUD’ can be associated with a person’s alcohol or illicit drug use qualifying for or needing treatment based on meeting diagnostic criteria. A person’s use and/or misuse of substances (inclusive of drugs and/or alcohol) is also important to consider as there are still interventions and services designed to reduce the harm associated with substance use and/or slow the progression of/development of SUD (22, 35). Thus, the remainder of the paper centers on the term substance mis/use and respective treatment. Typical treatment modalities for substance mis/use center non-violence however, the relationship between violence and SUD is less investigated (18, 27). Further, most treatment programs often preclude the absence of persons charged with violent offenses (36); this goes against what is known as best practice as past and recent research have highlighted the link between increased felony offense and decreases in drug treatment completion (37). This line of inquiry can help to dispel or understand why most consider victims to be under the influence while perpetrators are characterized as motivated by substances, along with other gendered and related identity stereotypes (38). A broad consideration of violence is utilized in this review, given the widespread influence on many biopsychosocial factors (39). This review considers intimate partner violence, sexual assault, stalking, child abuse, political and community violence, criminal violence, micro violence, and structural violence and oppression. A focus on treatment setting was chosen given the equivocal connection to relationships, violence, and offending, particularly for women (19, 20, 24, 30, 40).

The following research questions guide this study:

What are the common indicators of success or common outcomes tracked among studies testing the effects of treatment for people with substance mis/use (drugs and/or alcohol) with histories of violence?

What are the types of settings where people are receiving treatment?

Who is seeking treatment for their substance mis/use with histories of violence?

## 2 Methods

For this mapping review, initial test searches were performed in multiple databases (test searches included APA PsycInfo, Social Services Abstracts, Google Scholar and others) with a variety of search terms, trying to find sufficient coverage while also narrowing in on search terms that would properly locate the correct material. These initial searches revealed several aspects, detailed below, that influenced the design of the final search strategy.

### 2.1 Reviewing the literature

#### 2.1.1 Test Searches

First, there was significant overlap among different databases, and the subject journals being examined for the study were all represented in Web of Science’s collection. While a combination dataset from multiple databases may work for other studies, the scale of the eventual dataset meant that duplicative efforts would lead to unworkable amounts of data to sort through with minimal to no likelihood of additional relevant studies.

Second, there were significant obstacles in developing appropriately targeted language for the search. The study design focused on the overlap of exposure to violence and drug use, specifically addressing treatment options. However, there is a lack of controlled vocabulary for both halves of this topic. In various articles, exposure to violence was described as “victimization”, “interpersonal trauma”, “abuse”, or “IPV” among other terms. Similarly, drug use was described as “substance dependence”, “addiction”, or “SUD” or even indirectly with terms like “sober living home” or “detoxification” referencing the history of substance use. This variability in language made individual terms very difficult to nail down. Test searches frequently either missed test articles that should have been included or brought in significant amounts of material that was irrelevant to the study.

To address the combination of these two issues, a single database (Web of Science) was chosen for the focus of the search, and a wide net was cast, including several broad terms such as “drug-use” and “violence”. This led to a large initial dataset with many false positives, but a process of human annotation was used to narrow down the materials. Specific terms including alcohol as a separate search term were excluded due to this study’s focus on meeting criteria for substance misuse at large or SUD; essentially any use of substances that would warrant someone being referred to, enrolled in, and/or seeking treatment. Test searches including search terms specific to alcohol brought in many false positives that did not include alcohol use as a selection criterion. Test searches without alcohol as a separate search term brought in numerous studies related to disordered alcohol use that were considered for inclusion.

This study focused on research on treatment effectiveness. Our goal in examining where substance mis/use treatment is taking place (context) and who are enrolled in services was guided additionally by the focus on evidence-based evaluations. Since published material exists that describes treatment without any analysis, we centered studies with outcomes to get a focused sense of the field. To this end, while the initial search terms brought in many articles without a specific research/study focus, only articles that focused on specific outcomes were included in the final mapping analysis. Additionally, because treatment context is a study focus, the sampling of results conducted indicated that incorporating additional search terms did not increase information about study context.

#### 2.1.2 Study Selection

The publication inclusion dates were 2011-01-01 to 2022-01-01 and the following search string terms were used: (substance abuse OR use disorder OR drug-use OR substance use) AND (Violence OR partner abuse OR domestic abuse OR spous* abuse) AND (“Rct” or “rcts” or “randomized controlled trial” or “randomized controlled trial” or “outcome” or “intervention” or “interview*” or “measure” or “recruited” or “study” or “participants” or “self-report” or “analyzed” or “analyse*”) NOT adolescen*. This search was conducted in Web of Science and yielded an initial set of 8885 results, deduplication led to a search set of 8881 results for examination. All materials were examined in Rayyan QCRI. All results were reviewed by the first three authors and selected for inclusion or exclusion based on the following criteria. Inclusion criteria for the studies required that the articles must contain (a) outcome focused interventions related to an (b) adult patient population with a history of (c) exposure to violence *and* (d) substance mis/use. In the case of initial disagreements between authors, materials were discussed, and consensus was reached.

### 2.2 Inclusion criteria

#### 2.2.1 Population

All papers that focused on the adult population (18 and above) with a history of exposure to violence and substance mis/use were included. Studies located outside of the United States were also included given the universal experience of violence and substance mis/use; while each country is likely to have varying health and response systems to these issues, the limited knowledge to what extent these histories are addressed in populations seeking (or mandated to) treatment in the research calls for a wide review of available and eligible research. Studies not focusing on both histories for participants were excluded.

##### 2.2.1.1 History of substance mis/use/abuse^1^

This history includes any report of study participants with prior experience and report of drug/alcohol use or a diagnosed substance use disorder (SUD) or what is better referred to as substance misuse (35). The broad and inclusive search terms of use, abuse, and disorder regarding substances were used to capture anyone seeking treatment for either illicit drug or alcohol to seek broad understanding of its connection to histories of violence and treatment outcomes. Studies that did not clearly indicate the study population’s history of substance mis/use were excluded.

##### 2.2.1.2 History of exposure to violence

This history includes any report of study participants with prior or current exposure to violence that is inclusive of and moves beyond the standard inclusion criteria of intimate partner or domestic violence. The “violence” search term(s) were used to include the extant literature in the broad review while also including studies with participants living with substance mis/use with prior experiences with, but not limited to: war, combat, sex work, sex trafficking, sexual assault, varying abuse that may have been experienced in the home/from family members, community violence, and/or structural violence. Studies that did not clearly indicate the study population’s history of or exposure to violence were excluded. Studies included in this review also involved participants with post-traumatic stress disorder (PTSD), which indicates a direct exposure to or witnessing of a traumatic/violent experience [see American Psychiatric Association (41) for DSM-5 Diagnostic criteria for PTSD]. These studies were included as long as the trauma (PTSD) or violence exposure was defined in their inclusion criteria and also accompanying a report of current or past substance mis/use.

#### 2.2.2 Study design

The focus of this review was to identify who was seeking or mandated to treatment for substance mis/use with histories of violence, where they were receiving treatment, and the common indicators of success or outcomes from these studies. Thus, studies were included if they were a randomized controlled trial (RCT), pilot study, program evaluation, or any study examining the impact of a program or intervention published in peer-reviewed journals. Studies not involving the impact of treatment/programming/intervention on populations seeking treatment and reporting respective outcomes were excluded from this review (ex: cross-sectional designs; secondary analysis studies looking at a point in time, correlations, and/or relationships of variables to each other from secondary data). Secondary data analyses utilizing data from an RCT looking at the impact of an intervention on a given outcome that met the above criteria were also included.

### 2.3 Study selection and extraction

With the widely cast net, many articles were excluded from the final set of results, using standard exclusion tags to indicate why they were out of the scope of the study. As these tags were applied separately by all three authors, some were tagged for more than one exclusion criterion. 7,266 articles were marked as being for the wrong population, either not focused on adult patients, including persons using substances or not including individuals with a history of violence.1,730 articles were marked as wrong study design, not including any research element, with 459 tagged with wrong outcome, where the study’s intervention was not related to either drug use or violence.

Seventy-five articles were then reviewed in depth, with an additional 27 excluded for issues of study design, outcome, and population. The 48 articles included in the results were then examined for characteristics of the patient population, to evaluate the heterogeneity of different characteristics. Details of the studies included in this review can be found in the **Supplementary Table 1** at the end of this article. Characteristics that were examined include the participant relationship to violence (perpetrator, victim, or both), whether participants were parents, the use of cognitive behavioral therapy, the gender of participants, involvement in the criminal legal system, location of the study, treatment settings, and if treatment was specific to a certain substance. Based on these evaluative characteristics, Sankey^2^ diagram visualizations were created to explore the studies with a multi-dimensional approach, exploring how the intersection of multiple dimensions of identity and experience reveals gaps in the research. Sankey diagrams were chosen specifically for their ability to illustrate the breakdown of a larger set of results into smaller, more specific results. Segmenting out the dataset in this way demonstrates aspects of imbalance in research focus and gaps indicating future research. While Sankey flow diagrams have not been used for mapping reviews in the past, they were chosen here to establish the relationship between larger categories and to illustrate different ways in which the overall world of data can be analyzed.

## 3 Results

**Figure 1:**
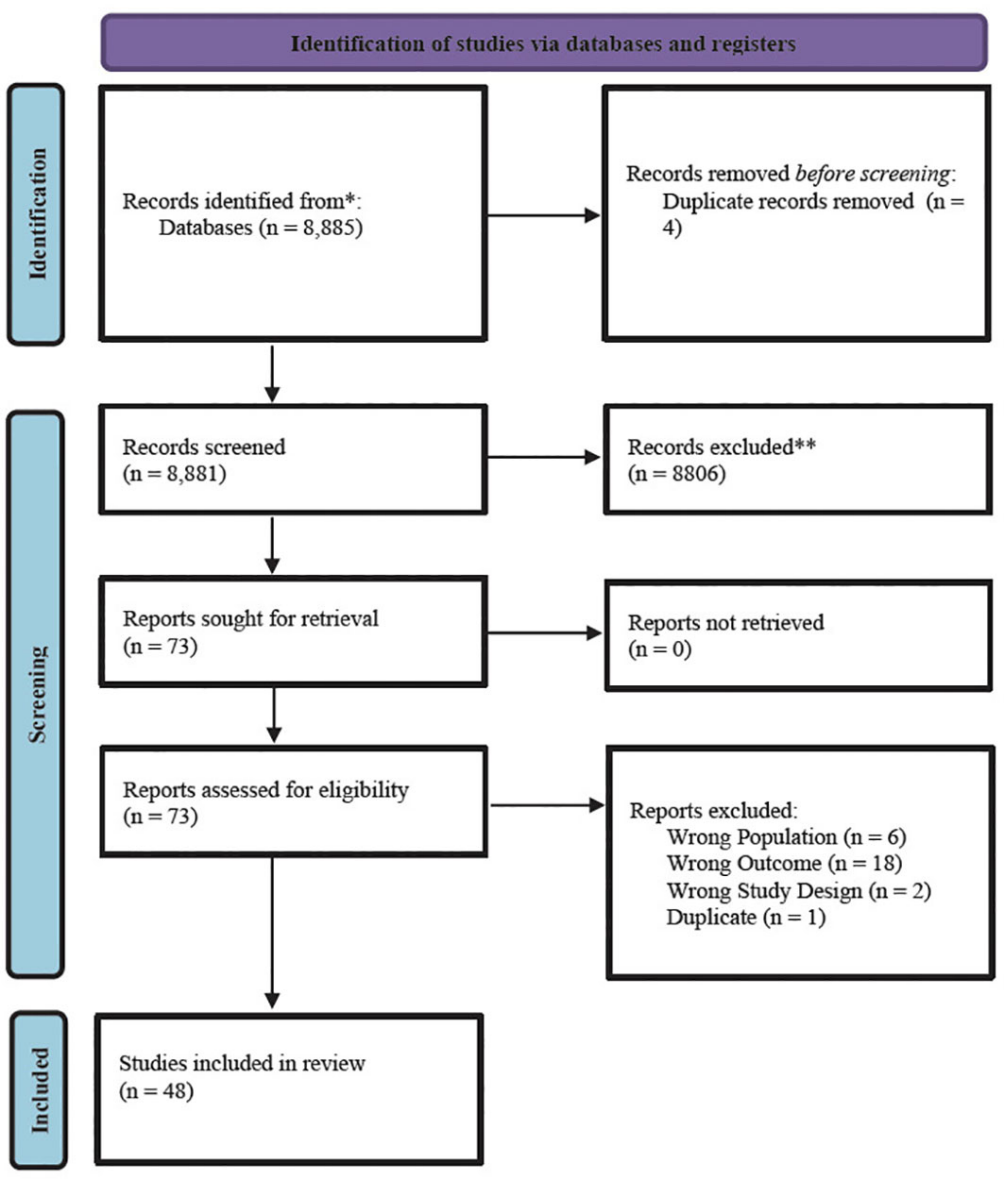
PRISMA flow diagram for substance use disorder and violence systematic mapping review. Figure from updated guidelines for reporting systematic reviews in Page et al. (42); *indicates the number of records identified from each database or register searched (rather than the total number across all databases/registers); **indicates the total number of records excluded by a human and by automation tools.

**Figure 2:**
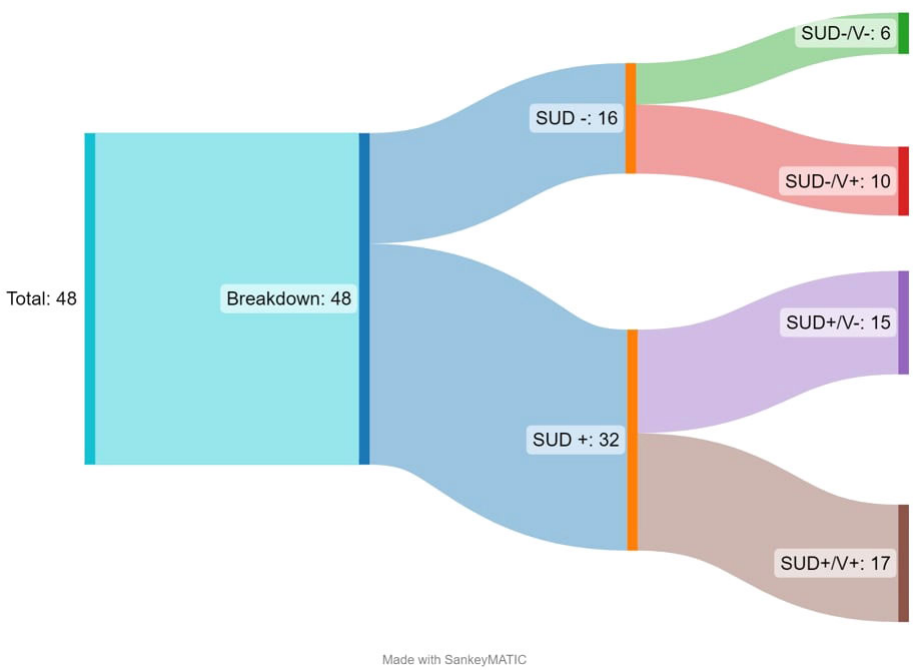
Sankey diagram illustrating breakdown of included studies by outcomes.

Of the 8,885 articles, 48 met inclusion criteria for this systematic mapping review. See **Figure 1** for the breakdown of inclusion and exclusion processes per the PRISMA guidelines (42). Most of the articles were RCTs or using secondary data from an RCT (n=34, 71%) and were based in the United States (n=33; 69%). Most studies included female identifying populations (n=19;40%), inclusive of the general population (not involved with the criminal legal system; n=33; 69%) and included the perpetrator of violence (or referred to as an offender in the study) as the treatment population (n=20, 42%). **Figure 2** is an initial breakdown of results demonstrating the disconnect between initial inclusion criteria and common outcomes of focus across the studies. While all studies included in the final review included exposure to violence and history of mis/using drugs/alcohol, this was not always reflected in the outcome focus of the studied interventions. Studies which had outcomes focused on addressing issues related to substance mis/use or SUD (n=32) are marked with SUD + (outcomes that *do not track* or document said variable will be noted by a minus sign ‘-’). This is further subdivided based on outcomes related to violence (V+; N=27). Among the selected studies, only 17 have outcomes related to both initial selection criteria, while 5 articles have outcomes related to neither of the initial selection criteria. While 48 studies may present a robust number of results, the variations in outcome focus show that there are significant variations in study density across the map.

The following results sections are specific to findings from the research questions looking at 1) the common indicators of success, 2) the treatment locations, and 3) identified characteristics of populations seeking treatment.

### 3.1 Common indicators of success

The most common indicators found across the 48 articles were trauma/PTSD symptoms, symptoms related to their drug/alcohol use, and experiences of violence (including IPV, perpetration, exposure to violent behavior.). **Table 1** depicts the list of common indicators/outcomes by study, while **Table 2** offers a breakdown of the types of violence experienced/reported in the included studies. While most studies were not specific to any particular substance and were inclusive of any reported mis/use of drugs and/or alcohol, the most commonly singled out substance was alcohol (n=12, 25%). Ten articles (21%) tracked symptoms associated with trauma/PTSD, however a much larger sample of articles tracked re-experiencing/perpetrating violence instead (only one article overlapped and tracked both; n=27, 56%). The most used indicator across the articles was tracking drug/alcohol use symptoms (n=32, 67%).

**Table 1:** Alphabetized list of included studies by outcome tracked.

Reference	Violence	Substance Use	Trauma/PTSD
Bohrman et al. (43)	X		
Catterall et al. (44)		X	
Chermack et al. (45		X	
Chermack et al. (46)		X	
Choo et al. (47)		X	
Coker et al. (48)		X	X
Deering et al. (49)	X	X	
Dheensa et al. (50)	X	X	
Easton et al. (51)	X	X	
Edwards et al. (52)		X	X
Edwards et al. (53)	X	X	
Empson et al. (54)		X	X
Fine et al. (55)	X	X	
Flanagan et al. (56)			
Gilbert et al. (57)	X	X	
Gilbert et al. (58)	X	X	
Gilchrist et al. (59)	X		
Grabbe et al. (60)			X
Harris and Hodges (61)	X		
Hershow et al. (62)	X		
Jones et al. (63	X	X	X
Kelley et al. (64)		X	
Kelley et al. (65)	X	X	
Kraanen et al. (66)	X	X	
Kubiak et al. (67)		X	X
LaPota et al. (68)	X	X	
Lee et al. (69)		X	X
Leight et al. (70)	X	X	
L'Engle et al. (71)	X	X	
Loόpez-Castro et al. (72)		X	X
Manhapra et al. (73)		X	X
Mbilinyi et al. (74)	X		
Murphy et al. (75)	X		
Parcesepe et al. (76)	X		
Reed et al. (77)		X	
Richter et al. (78)	X	X	
Satyanarayana et al. (79)	X		
Schiff et al. (80)			X
Schumm et al. (81)	X	X	
Schumm et al. (82)		X	X
Sevene et al. (83)	X		
Stover et al. (84)	X	X	
Stover et al. (85)	X	X	
Stover et al. (86)		X	
Stover et al. (87)	X	X	
Stuart et al. (88)	X		
Swopes et al. (89)		X	X
Tirado-Muñoz et al. (90)	X	X	

**Table 2:** The types of violence categories reported in the systematic mapping review.

Military Violence (n=4)	
Chermack et al. (46)	
Richter et al. (78)	
Manhapra et al. (73)	
Coker et al. (48)	
Sexual Violence (n=4)	
L'Engle et al. (71)	
Schiff et al. (80)	
Deering et al. (49)	
Parcesepe et al. (76)	
Partner Violence (n=24)	
Gilbert et al. (57)	
Dheensa et al. (50)	
Easton et al. (Easton et al. (51)	
Murphy et al. (75)	
Stover et al. (86)	
Gilchrist et al. (59)	
Satyanarayana et al. (79)	
Harris and Hodges (61)	
Leight et al. (70)	
Fine et al. (55)	
Flanagan et al. (56)	
Kraanen et al. (66)	
Stuart et al. (88)	
Schumm et al. (81)	
Kelly et al. (65)	
Stover et al. (84)	
Tirado-Muñoz et al. (90)	
Mbilinyi et al. (74)	
Sevene et al. (83)	
Stover et al. (85)	
Stover et al. (87)	
Bohrman et al. (43)	
Hershow et al. (62)	
Lee et al. (69)	
Non-Specified Violence (n=12)	
Kubiak et al. (67)	
Catterall et al. (44)	
Empson et al. (54)	
Ló́pez-Castro et al. (72)	
Edward et al. (Edward et al. (52)	
Gilbert et al. (58)	
Reed et al. (77)	
Swopes et al. (89)	
Choo et al. (47)	
Schumm et al. (82)	
Grabbe et al. (60)	
Chermack et al. (45)	
Childhood Violence (n=1)	
Kelley et al. (64)	
Sexual Violence & Partner Violence (n=2)	
Jones et al. (63)	
Edwards et al. (53)	
Childhood Violence & Partner Violence (n=1)	
LaPota et al. (68)	

### 3.2 The treatment locations

**Figure 3:**
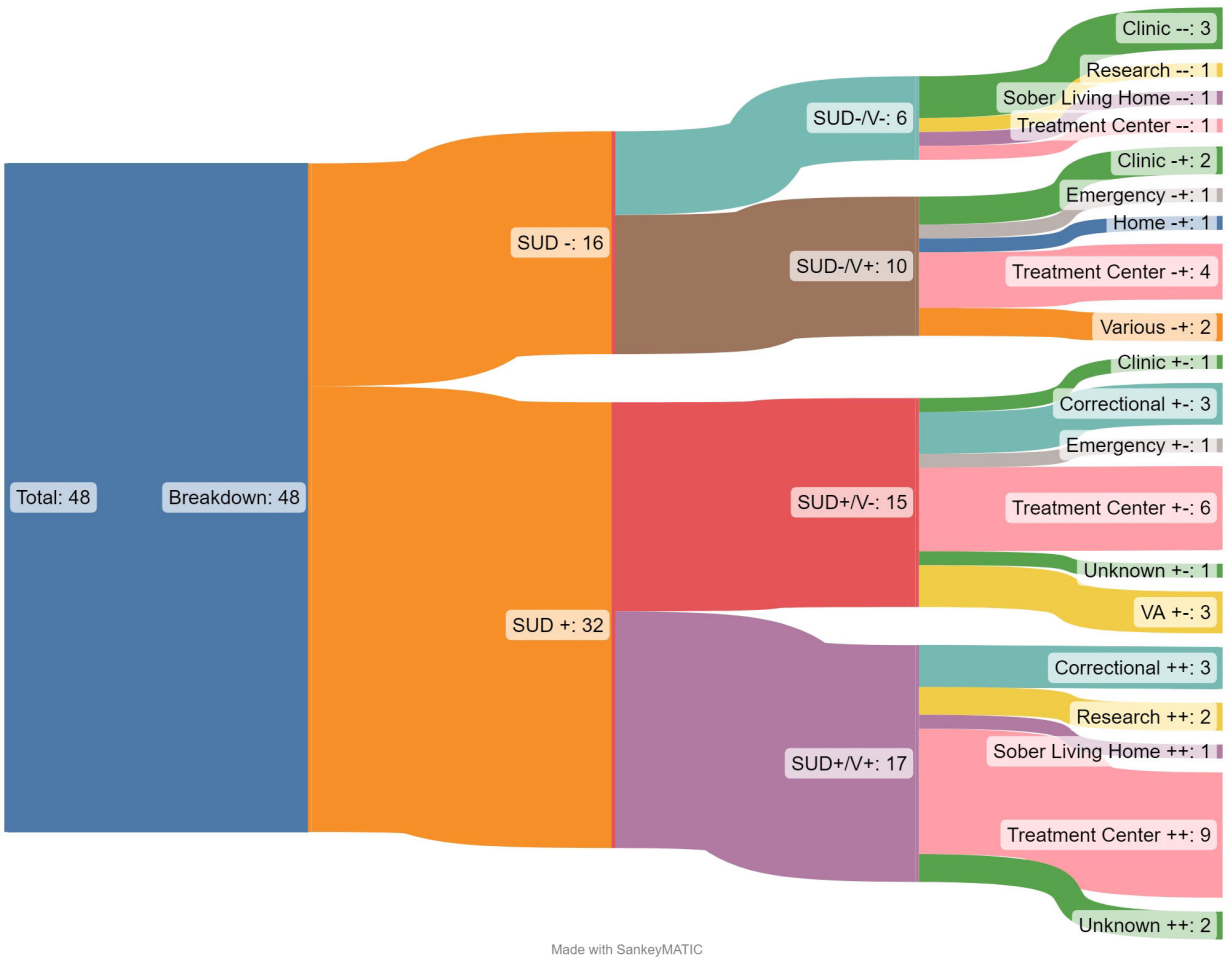
Sankey diagram of results by outcomes by treatment setting.

The average study was located within the United States (n=33; 69%) and was based in an outpatient program (n=35, 73%) located at a treatment center (n=20, 42%). While looking at where the treatment settings are located, we also noticed the interventions being utilized in these spaces could be characterized by those grounded in cognitive behavioral therapy (CBT) and those that were not. Most studies included in this review utilized a CBT- based intervention (n=28, 58%). Of the 15 studies (31%) located outside of the US, most occurred in treatment centers (n=6, 40%) or clinics (n=5, 33%). Additionally, the non-US based studies were predominately all based in outpatient settings (n=13; 87%) and were slightly more likely to use a non-CBT based intervention (n=8; 53%). **Figure 3** breaks down all studies first by SUD +/- and V+/- and then by the context of treatment. This shows that the settings of treatment vary widely in their distribution, with some contexts entirely absent from certain study permutations.

### 3.3 Identified characteristics of populations seeking treatment

**Figure 4:**
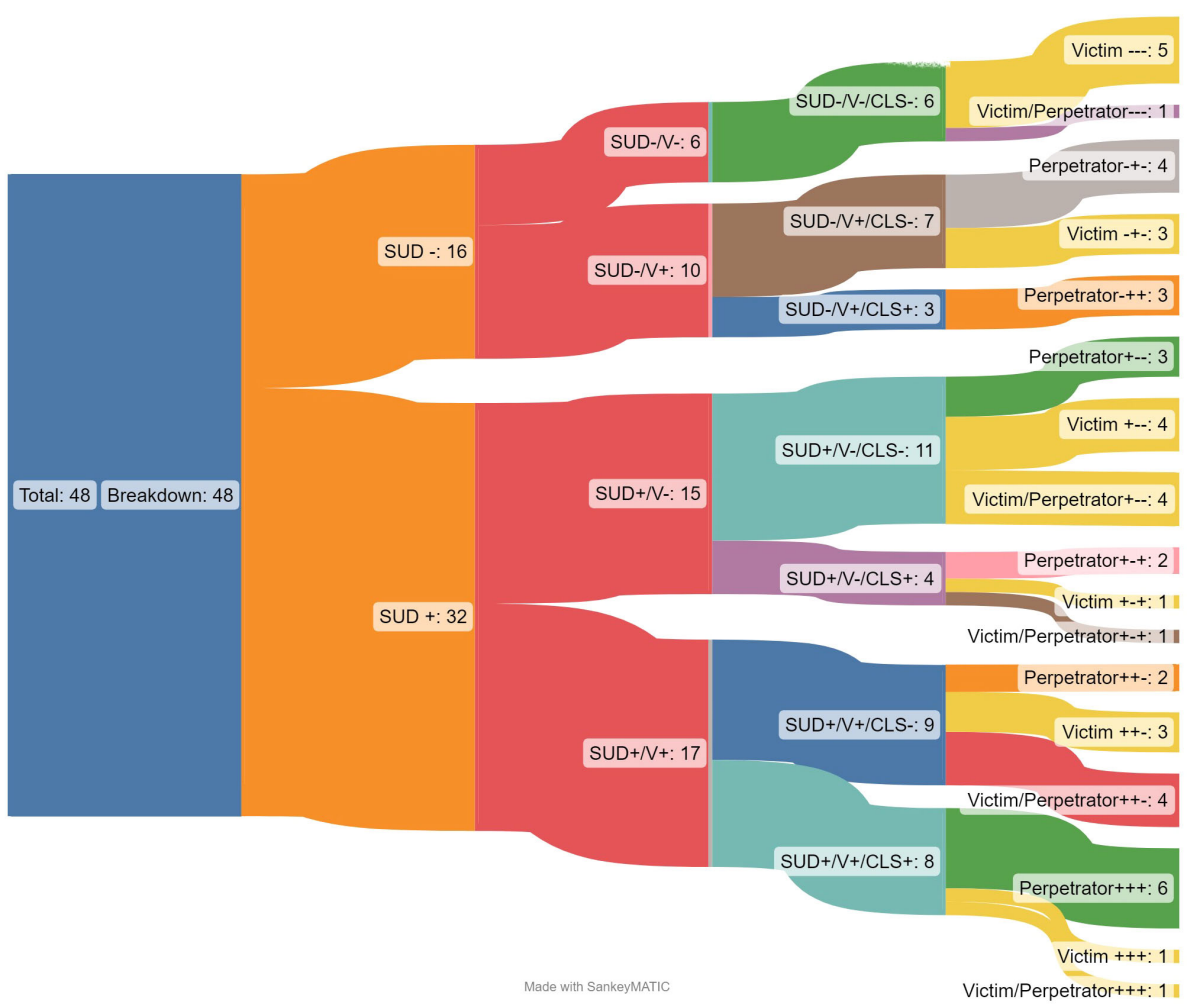
Sankey diagram by outcome and criminal legal setting by role in violent experience(s).

Forty percent of articles focused on female identifying participants (n=19;40%) with most studies studying interventions targeting populations labeled as the perpetrator of violent behavior (n=20; 50%). The most reported experience of violence in these studies included those concerning partner violence (n=24; 50%; see **Table 2**). The authors were attentive to studies that included sexual and gender minorities and discovered only three studies (6%) explicitly acknowledging inclusion of transgender participants or participants in relationship with partners of the same sex; three more studies (6%) were focused on participants with histories of or engaging in sex work. Only a small number more also included populations at risk for HIV or HIV seropositive (n=7, 15%). Four articles (8%) were specific to veteran populations; only one of these articles was specific to veterans exhibiting aggressive behavior, the remaining three were focusing on their trauma/PTSD symptoms. Relatedly, almost one-third of the studies (n=15; 31%) involved participants with arrest histories, under community corrections, and/or mandated to treatment for their violent offenses. **Figure 4** breaks down one of the most critical variable relationships within the studies, the division of study participants into the labels of victims or perpetrators of violence.

**Figure 5:**
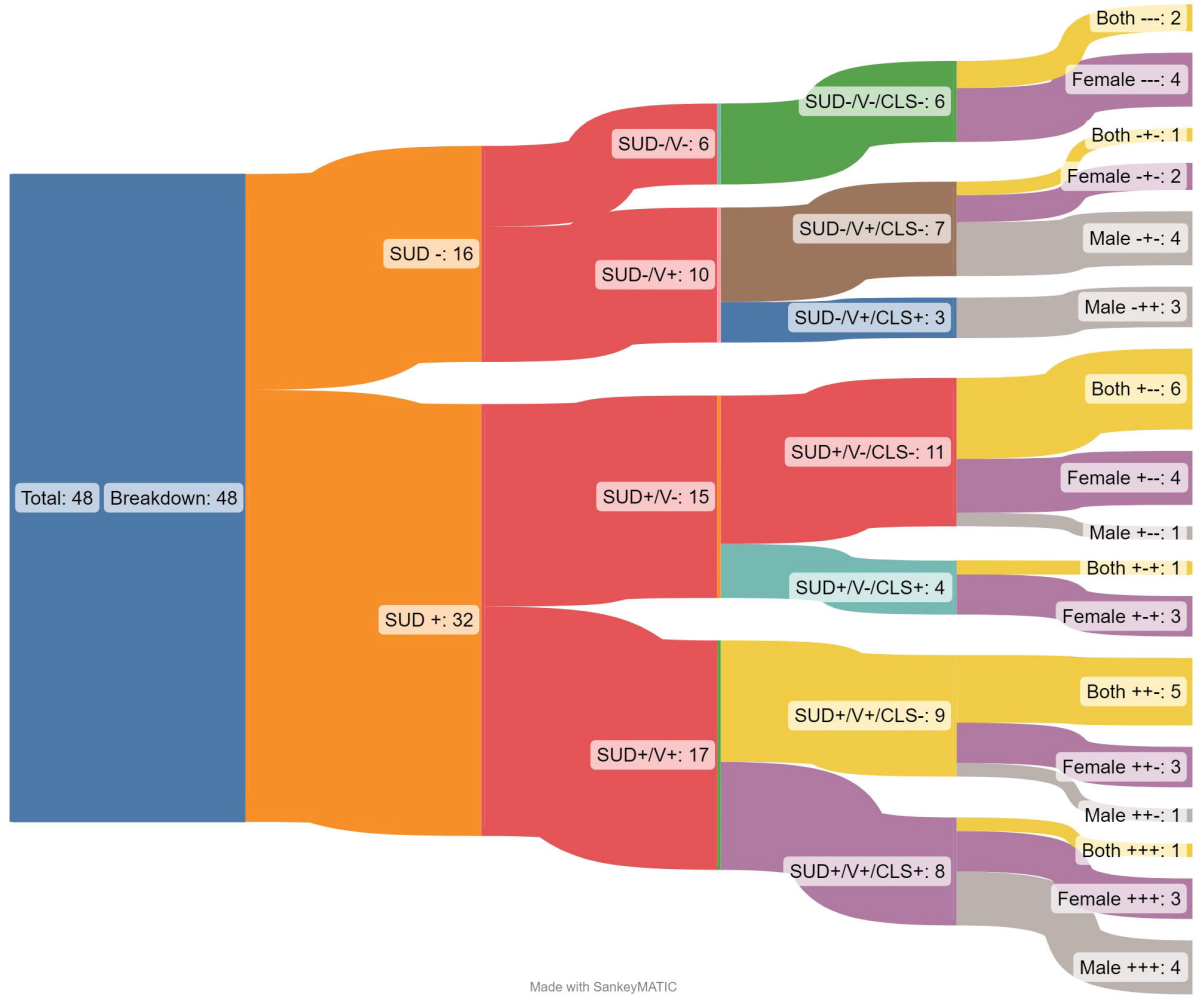
Sankey diagram by outcomes and criminal legal setting by gender of participants.

The articles including populations involved with the criminal legal system (n=15; 31%) tracked both outcome indicators specific to substance mis/use and violence. Further, all intervention studies focused on perpetrators of violence (n=20) predominantly in an outpatient setting (n= 16, 80%). Among the studies occurring in a correctional setting (predominantly community corrections, n=6), all studies tracked outcomes specific to their substance mis/use, but it was an even split for studies that also tracked outcomes related to experiences of violence. This descriptive finding highlights more attention to substance mis/use versus experiences of violence. Additionally, for studies involving populations involved with the criminal legal system (n=15) there were interesting outcome distinctions by gender variables. Among studies that tracked violence indicators, but not substance mis/use, they were all inclusive of male identifying populations (n=3). However, among these studies that tracked substance mis/use, but not violence exposure, all included female identifying population (n=4; one was inclusive of both genders). **Figure 5** breaks down studies within the criminal legal system by gender of participants, showing the divisions that emerge.

## 4 Discussion

This systematic mapping review offers preliminary insight into the extant literature on studies that have centered on serving individuals with substance mis/use and histories of violence. The results highlighted several key findings from the proposed research questions. First, the most common outcome/indicators of the intervention studies included PTSD symptoms, substance use, and experience with or contact with violence, with the most common outcome as substance mis/use. This is not surprising as one of the key inclusion criteria was searching for evidence involving participants seeking treatment for substance mis/use and has historically been one of the key primary outcomes in treatment research (91). However, in examining the articles and associated outcomes/indicators (see **Table 1**), there is inconsistent tracking of this primary outcome and only one of the articles tracked all three outcomes. We state this finding while also highlighting extant research documenting the link between substance mis/use and violence risk (92) and comorbidity with mental health disorders and traumatic experiences (such as adverse childhood experiences; 93).

Additionally, most studies occur in the United States and were delivered in treatment centers in an outpatient setting. This matches best practice per Substance Abuse Mental Health Services Administration (SAMHSA) in treatment SUDs and their comorbidities as it is recommended most populations experiencing complex treatment needs have a wide range of services available to them from more intensive, inpatient to less frequent, outpatient services that are typically offered in community-based treatment settings (94). However, with the rise in opioid use over the past decade, it is likely we will see treatment settings increasing within health clinics and more low-barrier harm reduction services in mobile health clinics to meet the high need of care alongside the growing rates of opioid use and overdoses (95). Relatedly, the Sankey diagram (**Figure 3**) showcases that there is high variability in treatment setting by outcomes tracked (experiences of violence and drug and alcohol mis/use), indicating several gaps in treatment that will be described in more detail in the next section.

An additional treatment characteristic finding of studies’ service participants with histories of violence seeking treatment for substance mis/use was that most used an intervention based in a cognitive behavioral therapy (CBT) approach; however, this was not as consistent in studies based outside the United States. This is a pertinent finding as evidence still suggests CBT approaches are still the “gold standard” within behavioral health and mental health service provision (96). Given the attention to special populations with marginalized identities in this review, we would be remiss if we did not state there is always room for improvement in any evidenced based treatment protocol, framework, and approach and, thus, effective providers must consider personal variables, including cultural traditions, beliefs, and values, in determining if CBT is an appropriate intervention. Ultimately, it is important to acknowledge most standards of care are based in Westernized ideologies and cultural norms and may not be relevant or effective as a standalone treatment option among Black, Indigenous, and other Populations of Color (97, 98).

Another finding from this review concerns who is seeking treatment and represented in the studies. Most of the studies involved female identifying populations and involved studies tailored to participants identifying as/labeled as the offender in the history of violence reported. These are two separate findings and, thus, do not suggest that most females were identifying as the perpetrators of violence; rather these findings highlight how females were the most common population to be included in studies or seeking treatment for substance mis/use with histories of trauma (often identifying as the survivor or victim) and male-identifying partners/populations usually identified as the offender and mostly represented in mandated treatment. The most prominent reported type of violence experienced by studies is partner violence (see **Table 2**). Given our broadened and inclusive conceptualization of *violence* as a search term, this review captured studies for populations reporting the following types of violence: military violence, sexual violence, childhood violence, sexual and partner violence, childhood and partner violence, and non-specified violence. The combination categories, while only represented by a small number of studies total (n=3, 63%), highlight the propensity for persons to experience more than one type of traumatic or violent event in their lifetime. This number could also be higher as certain studies may only have been targeting a) certain types of violent experiences, b) experiences occurring within a certain time frame and/or c) experiences occurring during a certain developmental period. The finding that partner violence is consistent with common focuses as most meta-analyses or systematic reviews focusing on violence and substance use disorders are largely on partner level or intimate relationship violence (99, 100). While partner violence is one of the more published comorbidities, it is critical to consider, assess, and integrate other categories of violence, both from a survivor and perpetrator standpoint as severity of substance use is linked to both exposure to violence at a personal (101) or community level (102).

### 4.1 Research and treatment gaps

In the early stages of forming the research questions, the authors had hypothesized that it was likely that populations receiving treatment in the included studies may not be particularly diverse and would not be attentive to specific types of racial or community level violence. We found this held true with most of the histories of violence were specific to that experienced within interpersonal relationships and micro-level violence. There were no qualifying articles that focused on histories of violence experienced within the community. Specifically, neighborhood gun violence and witnessing other traumatic events defined under the Adverse Childhood Experiences study (ACES; see 103), or other experiences of structural violence, including accumulated experiences with discrimination, prejudice, microaggressions, oppression within formal institutions including school, health, employment, and excessive use of force by police. Recent work by Lee and colleagues (104) found these types of community violence exposure as a distinct ACE grouping in their latent class analysis examining ACES and mental disorders in young adulthood.

Additionally, this is critical to working towards health equity in offering effective services for clients needing drug and alcohol treatment with histories of violence. Barrita and colleagues (28) conducted timely survey research elucidating the direct relationship between experiences of racial microaggressions and psychological distress and drug and alcohol mis/use. More research on experiences of violence within treatment can help illuminate the impact of microaggressions, or everyday acts of violence or assault, experienced in a space designed for healing (105). Researching microaggressions within the context of clients mandated to treatment and treatment within carceral systems has broad implications for violence conceptualizations (106); structural violence occurs in many different ubiquitous social systems. This line of research can advance understanding of structural violence as it can help identify institutional and systemic forms of violence experienced in healthcare, legal systems, and other entities and the groups and communities most impacted or targeted (see *racial macroaggressions* 105, 107). Additionally, only three articles included transgender or same sex couples indicating gender and sexually diverse populations as unrepresented within existing research; recent research highlights the intersectional impact of race and sexual identities and how treatment must incorporate and identify structural mechanisms, such as state and organizational level policies pertaining to antidiscrimination, in addition to individual level factors (108, 109).

Very few articles were explicit in their inclusion of parent populations (n=7, 15%). While this does not mean a broader parent population was not served within these studies, this is an important gap to address as we know that dependents witnessing acts of family violence committed in the home increase short and long-term developmental delays across cognitive and emotional domains (110) and can impact engagement in services (111). Additionally, there were almost no studies being conducted within correctional facilities, including jail or prison, despite over 60% of persons serving time in state prison have been convicted of a violent crime (112) and over 60% of persons being sentenced to jail meet criteria for a SUD (113); while it is unlikely that these programs are not happening in jail or prison settings, it is does show a gap in research that could prove helpful to programming offered to incarcerated persons given these percentages. This is most certainly the result of the inclusion of prisoners as a special population in human subjects research per guidance from the Office for Human Research Protections (OHRP); this additional level of oversight from Institutional Review Boards (IRB), however, are in place to protect vulnerable populations, inclusive of prisoners, from exploitation (eg, exposure to violence) in research. Recent work by Jones and colleagues (26) examining the long-term impact of intergenerational drug use and trauma among Black women involved with the criminal legal system found intergenerational substance use, experiences of trauma, witnessing/experiencing violence within and outside the family have more negative life outcomes (including continuing to use drugs and charges for child maltreatment); these findings underscore the importance of integrated care and the need for interventions accounting for intergenerational trauma and substance mis/use, such as incorporation of community level and life course approaches (114).

### 4.2 Limitations

There are several limitations to note for this systematic mapping review despite its contributions to the dearth of reviews focusing on a more inclusive view of violence in relation to substance mis/use. First, there is potential for some studies to have been missed due to the selection bias of studies that were reviewed and our inclusion criteria. The definition of special populations per OHRP and IRB, inclusive of prisoners, decreases the chance of virtually an entire special population from being better represented in a review of the evidence and minimizes populations that are exposed to/experience/perpetrate more violence and are also under more surveillance from police and correctional systems; thus, the results are not representative of this population despite several studies including populations with histories of criminal legal involvement.

The authors were intentional in locating search terms that would aid in the search for a broader and more representative sample of populations seeking/accessing care with histories of substance mis/use and violence. However, it needs to be noted for accurate interpretation of our results that the terms ‘treatment’ and ‘evidence-base’ are not synonymous and come with a heavy discourse. The terms ‘treatment’ or ‘services’ are used here as they are used in the various studies we included for this review; the articles included represent any study looking at the impact of the service, program, or treatment they were testing for patients seeking or mandated to services. This paper did not evaluate the quality of evidence, effectiveness of treatment, or examine the studies’ use of the term ‘treatment’; this could be a valuable systematic review in the future. Relatedly, the researchers were open to including experiences of structural violence among the participant histories. It, however, proved both too expansive and too elusive to accurately include within such a broad mapping survey; potential terms for structural violence did not appear as specific or prominent selection criterion for any of the studies examined. Thus, the conceptualization of structural violence within our inclusion criteria should be noted as a limitation and cannot be generalized to this important and distinct line of violence aimed for and/or experienced by this population.

Another limitation that is of critical importance to crisis care that has major overlap with populations witnessing and/or experiencing intimate, family, and/or community-based acts of violence is this review does not include or cover suicidality, attempts to suicide, or other acts of violence/harm to the self. Self-harm/violence and suicide are of utmost important to integrated care and effectively responding to substance mis/use, violence, and mental health services and should be considered in reading these results and proceeding with recommendations from research and treatment. Another note to consider in understanding results and drug and alcohol use, there was a variety of drug and alcohol use reported throughout the studies included in the review. These results are not connecting drug type or category to types of violence experienced and/or perpetrated, however this is a worthy line of research that can help understand risk propensity and effective programming by substance/drug of choice; for example, alcohol has an established connection and link to most charges of violent crime (115), yet recent research highlights stable (race or sex) and varying personal factors (income, housing, relationships) need to be considered with this finding to effectively understand this relationship (116). Overall, the intent of a systematic mapping review is broader in nature and results are not generalizable to treatment recommendations or the collective population of persons seeking care with histories of substance mis/use and violence.

## 5 Conclusion

This systematic mapping review examining the intersection of substance use treatment and histories of violence offers a foundational review from which other research and medical and mental health treatment providers can use to inform future recruitment, outreach strategy, and site considerations for implementation of integrated care for persons seeking care for their drug and alcohol use with histories of violence and trauma. This review highlighted common treatment settings, characteristics, and description of populations included in this line of research, however it is imperative to situate future research, practice, and policy in what we know, as well as what falls within the gap of knowledge. The reciprocal and bidirectional relationship between variables of drug and alcohol mis/use, violence, and trauma often begs the question of “which came first?” and sometimes, this question can be more easily answered for some clients and families than others. The findings of this review and associated research highlight that the service needs of persons using drugs and alcohol with histories of violence and trauma have complex care needs that will need to be simultaneously offered, addressed, and/or integrated within the myriad of treatment settings this special population may frequent. Integrated treatment needs also be considered alongside client and family level factors (eg, race, culture, religion, sexual orientation/preference, and other identity-level variables) and ensure relevant programming and services are offered in accessible and low-barrier treatment settings to strive for more equitable and responsive systems of care (117).

Part of a more equitable and responsive system of care is acknowledging that not everyone using alcohol/drugs needs help or wants help. While a person’s use may not qualify as a SUD per diagnostic criteria (DSM-5, 41), and thus does not want or qualify for treatment, we would be remiss if we did not acknowledge the potential impact of stigma in a person’s understanding of care and their want to seek help for substance mis/use (35, 118). To effectively respond to clients and families living with complex and interdependent needs, medical, mental health, and social service professionals must continually strive to understand and dissect personal bias and beliefs surrounding drug and alcohol use. Healthcare organizations and agencies working with the continuum of care of behavioral health services can also prioritize understanding and connecting with the community in where you practice to better understand the stigmas and gendered, normative, and Westernized stereotypes diverse and marginalized clients may experience when seeking help (119).

## Data availability statement

The raw data supporting the conclusions of this article will be made available by the authors, without undue reservation.

## Author contributions

SB: Conceptualization, Formal analysis, Investigation, Project administration, Supervision, Writing – original draft, Writing – review & editing. OG: Conceptualization, Investigation, Methodology, Writing – original draft, Writing – review & editing, Formal analysis. BA: Data curation, Formal analysis, Investigation, Methodology, Resources, Software, Supervision, Visualization, Writing – original draft, Writing – review & editing. AB: Conceptualization, Resources, Writing – review & editing, Writing – original draft.

## Footnotes

1 The authors recognize the terms ‘substance abuse’ and ‘disorder’ are stigmatizing terms and not representative of strengths-based terminology. We used these terms in our search given they are still used in the literature and make efforts in the results and discussion to use person-first language or reference substance mis/use (see 35 for more information).

2 All Sankey Diagrams were produced using the SankeyMatic tool at https://sankeymatic.com/.

## Supplementary material

Supplementary Table 1 Studies by outcomes, participant characteristics, and treatment studies.
