# Supplementary material for: Histories of violence among clients seeking substance use disorder treatment: a systematic mapping review
Source: Front Psychiatry. 2024 Mar 5;15:1307641. doi: 10.3389/fpsyt.2024.1307641 (PMC10948608; doi:10.3389/fpsyt.2024.1307641)
Supplement: Supplementary Table 1 — Studies by outcomes, participant characteristics, and treatment studies. [file DataSheet_1.pdf]

	Outcomes/Indicators				*Population Characteristics*							*Treatment Setting/Location Descriptions*			
**References**	**RCT**	**Trauma/PTSD**	**SUD**	**Violence tracking**	**Parents**	**Gender**	**Criminal Legal Involvement**	**Offender/Victim/Both**	**HIV**	**Substance**	**Other Characteristics (SS=Same Sex Couples Included)**	**use of CBT based intervention**	**Country**	**IP/OP**	**Location**
Bohrman, C., Tennille, J., Levin, K., Rodgers, M., & Rhodes, K. (2017). Being superwoman: Low income mothers surviving problem drinking and intimate partner violence. Journal of Family Violence, 32(7), 699-709. https://doi.org/10.1007/s10896-017-9932-5	Secondary	PTSD--	SUD--	V+	Mothers	female	CLI-	Victim	N	alcohol		CBT--	USA	Inpatient	emergency
Catterall, I., Mitchell, S. M., Dhingra, K., Conner, K. R., & Swogger, M. T. (2020). Brief motivational intervention for substance use may decrease violence among heavy alcohol users in a jail diversion program. Criminal Justice and Behavior, 48(3), 274-292. https://doi.org/10.1177/0093854820958747	Secondary	PTSD--	SUD+	V--	Not Parent Specific	female	CLI+	Victim and Offender	Y			CBT--	USA	Inpatient	Correctional
Chermack, S. T., Bonar, E. E., Goldstick, J. E., Winters, J., Blow, F. C., Friday, S., Ilgen, M. A., Rauch, S. A., Perron, B. E., Ngo, Q. M., & Walton, M. A. (2019). A randomized controlled trial for aggression and substance use involvement among veterans: Impact of combining motivational interviewing, cognitive behavioral treatment and telephone-based continuing care. Journal of Substance Abuse Treatment, 98, 78-88. https://doi.org/10.1016/j.jsat.2019.01.001	RCT+	PTSD--	SUD+	V--	Not Parent Specific	both	CLI-	Victim and Offender	N		veterans	CBT +	USA	Outpatient	VA
Chermack, S. T., Bonar, E. E., Ilgen, M. A., Walton, M. A., Cunningham, R. M., Booth, B. M., & Blow, F. C. (2016). Developing an integrated violence prevention for men and women in treatment for substance use disorders. Journal of Interpersonal Violence, 32(4), 581-603. https://doi.org/10.1177/0886260515586369	RCT+	PTSD--	SUD+	V--	Not Parent Specific	both	CLI-	Offender	N			CBT +	USA	Both	Treatment Center
Choo, E. K., McGregor, A. J., Mello, M. J., & Baird, J. (2013). Gender, violence and brief interventions for alcohol in the emergency department. Drug and Alcohol Dependence, 127(1-3), 115-121. https://doi.org/10.1016/j.drugalcdep.2012.06.021	Secondary	PTSD--	SUD+	V--	Not Parent Specific	both	CLI-	Victim and Offender	N	alcohol		CBT--	USA	Inpatient	Emergency
Coker, K. L., Stefanovics, E., & Rosenheck, R. (2016). Correlates of improvement in substance abuse among dually diagnosed veterans with post-traumatic stress disorder in specialized intensive VA treatment. Psychological Trauma: Theory, Research, Practice, and Policy, 8(1), 41-48. https://doi.org/10.1037/tra0000061	Secondary	PTSD+	SUD+	V--	Not Parent Specific	both	CLI-	Victim and Offender	N		veterans	CBT--	USA	Outpatient	VA
Deering, K. N., Kerr, T., Tyndall, M. W., Montaner, J. S., Gibson, K., Irons, L., & Shannon, K. (2011). A peer-led mobile outreach program and increased utilization of detoxification and residential drug treatment among female sex workers who use drugs in a Canadian setting. Drug and Alcohol Dependence, 113(1), 46-54. https://doi.org/10.1016/j.drugalcdep.2010.07.007	RCT--	PTSD--	SUD--	V--	Not Parent Specific	female	CLI-	Victim	N		sex work	CBT--	Canada	Outpatient	Clinic
Dheensa, S., Halliwell, G., Johnson, A., Henderson, J., Love, B., Radcliffe, P., Gilchrist, L., & Gilchrist, G. (2021). Perspectives on motivation and change in an intervention for men who use substances and perpetrate intimate partner abuse: Findings from a qualitative evaluation of the advance intervention. Journal of Interpersonal Violence, 37(15-16), NP13342-NP13372. https://doi.org/10.1177/0886260521997436	RCT--	PTSD--	SUD+	V+	Not Parent Specific	male	CLI+	Offender	N			CBT +	UK	Outpatient	Treatment Center
Easton, C. J., Crane, C. A., & Mandel, D. (2017). A randomized controlled trial assessing the efficacy of cognitive behavioral therapy for substance-dependent domestic violence offenders: An integrated substance abuse-domestic violence treatment approach (<scp>SADV</scp>). Journal of Marital and Family Therapy, 44(3), 483-498. https://doi.org/10.1111/jmft.12260	RCT--	PTSD--	SUD+	V--	Both Parents	both	CLI-	Offender	N			CBT +	USA	Outpatient	Treatment Center
Edwards, K. M., Haynes, E. E., Palmer, K. M., & Murphy, S. (2017). Sense of community among female residents of a trauma-informed sober living home. Substance Use & Misuse, 53(6), 1051-1055. https://doi.org/10.1080/10826084.2017.1385629	RCT--	PTSD--	SUD--	V--	Not Parent Specific	both	CLI-	Victim	N			CBT +	USA	Inpatient	Sober Living Home

Edwards, K. M., Murphy, S., Palmer, K. M., Haynes, E. E., Chapo, S., Ekdahl, B. A., & Buel, S. (2017). Co-occurrence of and recovery from substance abuse and lifespan victimization: A qualitative study of female residents in trauma-informed sober living homes. Journal of Psychoactive Drugs, 49(1), 74-82. https://doi.org/10.1080/02791072.2016.1273566	RCT--	PTSD--	SUD+	V+	Not Parent Specific	female	CLI-	Victim	N			CBT +	USA	Inpatient	Sober Living Home
Empson, S., Cuca, Y. P., Cocohoba, J., Dawson-Rose, C., Davis, K., & Machtinger, E. L. (2017). Seeking safety group therapy for co-occurring substance use disorder and PTSD among transgender women living with HIV: A pilot study. Journal of Psychoactive Drugs, 49(4), 344-351. https://doi.org/10.1080/02791072.2017.1320733	RCT--	PTSD+	SUD+	V--	Not Parent Specific	female	CLI-	Victim	Y		transgender	CBT +	USA	Outpatient	Clinic
Fine, S. L., Kane, J. C., Murray, S. M., Skavenski, S., Paul, R., & Murray, L. K. (2021). Moderator effects in a randomized controlled trial of the common elements treatment approach (CETA) for intimate partner violence and hazardous alcohol use in Zambia. Drug and Alcohol Dependence, 228, 108995. https://doi.org/10.1016/j.drugalcdep.2021.108995	RCT+	PTSD--	SUD+	V+	Not Parent Specific	both	CLI-	Victim and Offender	N	alcohol		CBT +	Zambia	Outpatient	Unknown
Flanagan, J. C., Jarnecke, A. M., Leone, R. M., & Oesterle, D. W. (2020). Effects of couple conflict on alcohol craving: Does intimate partner violence play a role? Addictive Behaviors, 109, 106474. https://doi.org/10.1016/j.addbeh.2020.106474	RCT+	PTSD--	SUD--	V--	Not Parent Specific	both	CLI-	Victim and Offender	N	alcohol		CBT--	USA	Outpatient	Research
Gilbert, L., Goddard-Eckrich, D., Hunt, T., Ma, X., Chang, M., Rowe, J., McCrimmon, T., Johnson, K., Goodwin, S., Almonte, M., & Shaw, S. A. (2016). Efficacy of a computerized intervention on HIV and intimate partner violence among substance-using women in community corrections: A randomized controlled trial. American Journal of Public Health, 106(7), 1278-1286. https://doi.org/10.2105/ajph.2016.303119	RCT+	PTSD--	SUD+	V+	Not Parent Specific	female	CLI+	Victim	Y			CBT--	USA	Inpatient	Correctional
Gilbert, L., Shaw, S. A., Goddard-Eckrich, D., Chang, M., Rowe, J., McCrimmon, T., Almonte, M., Goodwin, S., & Epperson, M. (2015). Project WINGS (Women initiating new goals of safety): A randomised controlled trial of a screening, brief intervention and referral to treatment (SBIRT) service to identify and address intimate partner violence victimisation among substance-using women receiving community supervision. Criminal Behaviour and Mental Health, 25(4), 314-329. https://doi.org/10.1002/cbm.1979	RCT+	PTSD--	SUD+	V+	Not Parent Specific	female	CLI+	Victim and Offender	N		SS	CBT--	USA	Outpatient	Correctional
Gilchrist, G. (2021). Perspectives on motivation and change in an intervention for men who use substances and perpetrate intimate partner abuse: Findings from a qualitative evaluation of the advance intervention. Journal of Interpersonal Violence, 37(15-16), NP13342-NP13372. https://doi.org/10.1177/0886260521997436	RCT+	PTSD--	SUD+	V+	Not Parent Specific	both	CLI+	Offender	N			CBT +	USA	Outpatient	Treatment Center
Gilchrist, G., Potts, L., Radcliffe, P., Halliwell, G., Dheensa, S., Henderson, J., Johnson, A., Love, B., Gilchrist, E., Feder, G., Parrott, S., Li, J., McMurran, M., Kirkpatrick, S., Stephens-Lewis, D., Easton, C., Berbary, C., & Landau, S. (2021). Advance integrated group intervention to address both substance use and intimate partner abuse perpetration by men in substance use treatment: A feasibility randomised controlled trial. BMC Public Health, 21(1). https://doi.org/10.1186/s12889-021-11012-3	RCT+	PTSD--	SUD--	V+	Not Parent Specific	male	CLI+	Offender	N			CBT +	UK	Outpatient	Treatment Center
Grabbe, L., Higgins, M., Jordan, D., Noxsel, L., Gibson, B., & Murphy, J. (2020). The community resiliency model®: A pilot of an Interoception intervention to increase the emotional self-regulation of women in addiction treatment. International Journal of Mental Health and Addiction, 19(3), 793-808. https://doi.org/10.1007/s11469-019-00189-9	RCT--	PTSD--	SUD--	V--	Not Parent Specific	female	CLI-	Victim	N			CBT--	USA	Outpatient	Treatment Center

Harris, L., & Hodges, K. (2019). Responding to complexity: Improving service provision for survivors of domestic abuse with 'complex needs'. Journal of Gender-Based Violence, 3(2), 167-184. https://doi.org/10.1332/239868019x15538587319964	RCT--	PTSD--	SUD--	V+	Not Parent Specific	both	CLI-	Victim	N			CBT--	UK	Outpatient	Various
Hershow, R. B., Reyes, H. L., Ha, T. V., Chander, G., Mai, N. V., Sripaipan, T., Frangakis, C., Dowdy, D. W., Latkin, C., Hutton, H. E., Pettifor, A., Maman, S., & Go, V. F. (2020). Longitudinal analysis of alcohol use and intimate partner violence perpetration among men with HIV in northern Vietnam. Drug and Alcohol Dependence, 213, 108098. https://doi.org/10.1016/j.drugalcdep.2020.108098	Secondary	PTSD--	SUD--	V+	Not Parent Specific	male	CLI-	Offender	Y	alcohol		CBT +	Vietnam	Outpatient	Clinic
Jones, D. L., Kashy, D., Villar-Loubet, O. M., Cook, R., & Weiss, S. M. (2012). The impact of substance use, sexual trauma, and intimate partner violence on sexual risk intervention outcomes in couples: A randomized trial. Annals of Behavioral Medicine, 45(3), 318-328. https://doi.org/10.1007/s12160-012-9455-5	RCT+	PTSD+	SUD+	V+	Not Parent Specific	both	CLI-	Victim and Offender	Y	alcohol		CBT +	USA	Outpatient	Research
Kelly, L. M., Crane, C. A., Zajac, K., & Easton, C. J. (2021). The impact of depressive symptoms on response to integrated cognitive behavioral therapy for substance use disorders and intimate partner violence. Advances in Dual Diagnosis, 14(3), 85-98. https://doi.org/10.1108/add-09-2020-0020	Secondary	PTSD--	SUD+	V+	Not Parent Specific	male	CLI+	Offender	N			CBT +	USA	Outpatient	Treatment Center
Kraanen, F. L., Vedel, E., Scholing, A., & Emmelkamp, P. M. (2013). The comparative effectiveness of integrated treatment for substance abuse and partner violence (I-stop) and substance abuse treatment alone: A randomized controlled trial. BMC Psychiatry, 13(1). https://doi.org/10.1186/1471-244x-13-189	RCT+	PTSD--	SUD+	V+	Not Parent Specific	both	CLI-	Offender	N			CBT +	Netherlands	Outpatient	Treatment Center
Kubiak, S., Fedock, G., Kim, W. J., & Bybee, D. (2016). Long-term outcomes of a RCT intervention study for women with violent crimes. Journal of the Society for Social Work and Research, 7(4), 661-679. https://doi.org/10.1086/689356	RCT+	PTSD+	SUD+	V--	Not Parent Specific	female	CLI+	Offender	N			CBT +	USA	Inpatient	Correctional
LaPota, H. B., Donohue, B., Warren, C. S., & Allen, D. N. (2011). Incorporating a healthy living curriculum within family behavior therapy: A clinical case example in a woman with a history of domestic violence, child neglect, drug abuse, and obesity. Journal of Family Violence, 26(3), 227-234. https://doi.org/10.1007/s10896-011-9358-4	RCT--	PTSD--	SUD+	V+	Mothers	female	CLI+	Offender	N			CBT +	USA	Outpatient	Correctional
Lee, M. Y., Eads, R., & Hoffman, J. (2021). "I felt it and I let it go": Perspectives on meditation and emotional regulation among female survivors of interpersonal trauma with co-occurring disorders. Journal of Family Violence, 37(4), 629-641. https://doi.org/10.1007/s10896-021-00329-7	RCT--	PTSD+	SUD+	V--	Not Parent Specific	female	CLI-	Victim	N			CBT--	USA	Inpatient	Treatment Center
Leight, J., Deyessa, N., Verani, F., Tewolde, S., & Sharma, V. (2020). An intimate partner violence prevention intervention for men, women, and couples in Ethiopia: additional findings on substance use and depressive symptoms from a cluster-randomized controlled trial. PLoS medicine, 17(8), e1003131.	RCT+	PTSD--	SUD+	V+	Not Parent Specific	both	CLI-	Victim and Offender	N			CBT--	Ethiopia	Outpatient	Unknown
L'Engle, K. L., Mwarogo, P., Kingola, N., Sinkele, W., & Weiner, D. H. (2014). A randomized controlled trial of a brief intervention to reduce alcohol use among female sex workers in Mombasa, Kenya. JAIDS Journal of Acquired Immune Deficiency Syndromes, 67(4), 446-453. https://doi.org/10.1097/qai.0000000000000335	RCT+	PTSD--	SUD--	V--	Not Parent Specific	female	CLI-	Victim	N	Alcohol	sex work	CBT--	Kenya	Outpatient	Clinic
López-Castro, T., Smith, K. Z., Nicholson, R. A., Armas, A., & Hien, D. A. (2019). Does a history of violent offending impact treatment response for comorbid PTSD and substance use disorders? A secondary analysis of a randomized controlled trial. Journal of Substance Abuse Treatment, 97, 47-58. https://doi.org/10.1016/j.jsat.2018.11.009	RCT+	PTSD+	SUD+	V--	Not Parent Specific	both	CLI+	Offender	N			CBT +	USA	Outpatient	Treatment Center

Manhapra, A., Stefanovics, E., & Rosenheck, R. (2015). Treatment outcomes for veterans with PTSD and substance use: Impact of specific substances and achievement of abstinence. Drug and Alcohol Dependence, 156, 70-77. https://doi.org/10.1016/j.drugalcdep.2015.08.036	RCT--	PTSD+	SUD+	V--	Not Parent Specific	both	CLI-	Victim and Offender	N		veterans	CBT--	USA	Outpatient	VA
Mbilinyi, L. F., Neighbors, C., Walker, D. D., Roffman, R. A., Zegree, J., Edleson, J., & O'Rourke, A. (2010). A telephone intervention for substance-using adult male perpetrators of intimate partner violence. Research on Social Work Practice, 21(1), 43-56. https://doi.org/10.1177/1049731509359008	RCT+	PTSD--	SUD--	V+	Not Parent Specific	male	CLI-	Offender	N		SS	CBT--	USA	Outpatient	Home
Murphy, C. M., Ting, L. A., Jordan, L. C., Musser, P. H., Winters, J. J., Poole, G. M., & Pitts, S. C. (2018). A randomized clinical trial of motivational enhancement therapy for alcohol problems in partner violent men. Journal of Substance Abuse Treatment, 89, 11-19. https://doi.org/10.1016/j.jsat.2018.03.004	RCT+	PTSD--	SUD--	V+	Not Parent Specific	male	CLI-	Offender	N	alcohol		CBT--	USA	Outpatient	Treatment Center
Parcesepe, A. M., L'Engle, K. L., Martin, S. L., Green, S., Sinkele, W., Suchindran, C., Speizer, I. S., Mwarogo, P., & Kingola, N. (2016). The impact of an alcohol harm reduction intervention on interpersonal violence and engagement in sex work among female sex workers in Mombasa, Kenya: Results from a randomized controlled trial. Drug and Alcohol Dependence, 161, 21-28. https://doi.org/10.1016/j.drugalcdep.2015.12.037	RCT+	PTSD--	SUD--	V+	Not Parent Specific	female	CLI-	Victim	N	alcohol	sex work	CBT--	Kenya	Outpatient	Clinic
Reed, E., Myers, B., Novak, S. P., Browne, F. A., & Wechsberg, W. M. (2014). Experiences of violence and association with decreased drug abstinence among women in Cape Town, South Africa. AIDS and Behavior, 19(1), 192-198. https://doi.org/10.1007/s10461-014-0820-1	RCT+	PTSD--	SUD+	V--	Not Parent Specific	female	CLI-	Victim	Y			CBT--	South Africa	Outpatient	Unknown
Richter, A., Sason, A., Adelson, M., Frish, O., & Peles, E. (2020). Cognitive state, substance use patterns and outcome after discharge from Kfar Izun, a unique rehabilitation facility. Journal of Addictive Diseases, 38(4), 387-399.	RCT--	PTSD--	SUD+	V+	Not Parent Specific	both	CLI-	Victim and Offender	N		veterans	CBT--	Israel	Inpatient	Treatment Center
Satyanarayana, V. A., Nattala, P., Selvam, S., Pradeep, J., Hebbani, S., Hegde, S., & Srinivasan, K. (2016). Integrated cognitive behavioral intervention reduces intimate partner violence among alcohol dependent men, and improves mental health outcomes in their spouses: A clinic based randomized controlled trial from South India. Journal of Substance Abuse Treatment, 64, 29-34. https://doi.org/10.1016/j.jsat.2016.02.005	RCT+	PTSD--	SUD--	V+	Not Parent Specific	male	CLI-	Offender	N	alcohol		CBT +	India	Inpatient	Treatment Center
Schiff, M., Nacasch, N., Levit, S., Katz, N., & Foa, E. B. (2015). Prolonged exposure for treating PTSD among female methadone patients who were survivors of sexual abuse in Israel. Social Work in Health Care, 54(8), 687-707. https://doi.org/10.1080/00981389.2015.1058311	RCT+	PTSD+	SUD--	V--	Not Parent Specific	female	CLI-	Victim	Y	methadone		CBT--	Israel	Outpatient	Clinic
Schumm, J. A., O'Farrell, T. J., Murphy, M. M., & Muchowski, P. (2018). Partner violence among drug-abusing women receiving behavioral couples therapy versus individually-based therapy. Journal of Substance Abuse Treatment, 92, 1-10. https://doi.org/10.1016/j.jsat.2018.06.004	Secondary	PTSD--	SUD+	V+	Not Parent Specific	female	CLI-	Victim	N			CBT +	USA	Outpatient	Treatment Center
Schumm, J. A., O'Farrell, T. J., Murphy, M. M., & Muchowski, P. (2019). Efficacy of behavioral couples therapy versus individual recovery counseling for addressing posttraumatic stress disorder among women with drug use disorders. Journal of Traumatic Stress, 32(4), 595-605. https://doi.org/10.1002/jts.22415	Secondary	PTSD+	SUD+	V--	Not Parent Specific	female	CLI-	Victim	N			CBT +	USA	Outpatient	Treatment Center
Sevene, A. M., Edlund, J. E., & Easton, C. J. (2017). Role of offender perception in treatment outcome. Advances in Dual Diagnosis, 10(2), 83-94. https://doi.org/10.1108/add-01-2017-0003	RCT+	PTSD--	SUD--	V+	Not Parent Specific	male	CLI+	Offender	N			CBT +	USA	Outpatient	Treatment Center
Stover, C. S. (2015). Fathers for change for substance use and intimate partner violence: Initial community pilot. Family Process, 54(4), 600-609. https://doi.org/10.1111/famp.12136	RCT+	PTSD--	SUD+	V+	Fathers	male	CLI+	Offender	N			CBT +	USA	Outpatient	Research
Stover, C. S., Carlson, M., & Patel, S. (2017). Integrating intimate partner violence and parenting intervention into residential substance use disorder treatment for fathers. Journal of Substance Abuse Treatment, 81, 35-43. https://doi.org/10.1016/j.jsat.2017.07.013	RCT--	PTSD--	SUD+	V+	Fathers	male	CLI-	Offender	N			CBT +	USA	Outpatient	Treatment Center

Stover, C. S., McMahon, T. J., & Easton, C. (2010). The impact of fatherhood on treatment response for men with co-occurring alcohol dependence and intimate partner violence. The American Journal of Drug and Alcohol Abuse, 37(1), 74-78. https://doi.org/10.3109/00952990.2010.535585	Secondary	PTSD--	SUD+	V+	Fathers	male	CLI+	Offender	N	alcohol	CBT +	USA	Outpatient	Treatment Center
Stover, C. S., McMahon, T. J., & Moore, K. (2019). A randomized pilot trial of two parenting interventions for fathers in residential substance use disorder treatment. Journal of Substance Abuse Treatment, 104, 116-127. https://doi.org/10.1016/j.jsat.2019.07.003	RCT+	PTSD--	SUD+	V--	Fathers	male	CLI-	Offender	N		CBT +	USA	Inpatient	Treatment Center
Stuart, G. L., Shorey, R. C., Moore, T. M., Ramsey, S. E., Kahler, C. W., O'Farrell, T. J., Strong, D. R., Temple, J. R., & Monti, P. M. (2013). Randomized clinical trial examining the incremental efficacy of a 90-minute motivational alcohol intervention as an adjunct to standard batterer intervention for men. Addiction, 108(8), 1376-1384. https://doi.org/10.1111/add.12142	RCT+	PTSD--	SUD--	V+	Not Parent Specific	male	CLI+	Offender	N	alcohol	CBT +	USA	Outpatient	Various
Swopes, R. M., Davis, J. L., & Scholl, J. A. (2016). Treating substance abuse and trauma symptoms in incarcerated women. Journal of Interpersonal Violence, 32(7), 1143-1165. https://doi.org/10.1177/0886260515587668	RCT--	PTSD+	SUD+	V--	Not Parent Specific	female	CLI+	Victim	N		CBT +	USA	Inpatient	Correctional
Tirado-Muñoz, J., Gilchrist, G., Lligoña, E., Gilbert, L., & Torrens, M. (2015). A group intervention to reduce intimate partner violence among female drug users. Results from a randomized controlled pilot trial in a community substance-abuse center. Adicciones, 27(3), 168-178.	RCT+	PTSD--	SUD+	V+	Not Parent Specific	female	CLI-	Victim	N		CBT +	Spain	Outpatient	Treatment Center
